# Expertise Affects Inter-Observer Agreement at Peripheral Locations within a Brain Tumor

**DOI:** 10.3389/fpsyg.2017.01628

**Published:** 2017-09-20

**Authors:** Emily M. Crowe, William Alderson, Jonathan Rossiter, Christopher Kent

**Affiliations:** ^1^School of Experimental Psychology, University of Bristol Bristol, United Kingdom; ^2^Department of Engineering Mathematics, University of Bristol Bristol, United Kingdom

**Keywords:** brain tumor, novice-expert differences, tumor delineation, medical image perception, radiological diagnosis

## Abstract

Magnetic resonance imaging (MRI) is a crucial tool for clinical brain tumor detection and delineation. Since the process of gross tumor volume delineation resides with clinicians, a better understanding of how they perform this task is required if improvements in life expectancy are to be made. Novice-expert comparison studies have been used to examine the effect of expertise on abnormality detection, but little research has investigated expertise-related differences in brain tumor delineation. In this study, undergraduate students (novices) and radiologists (experts) inspected a combination of T1 and T2 single and whole brain MRI scans, each containing a tumor. Using a tablet and stylus to provide an interactive environment, participants had an unlimited amount of time to scroll freely through the MRI slices and were instructed to delineate (i.e., draw a boundary) around any tumorous tissue. There was no reliable evidence for a difference in the gross tumor volume or total number of slices delineated between experts and novices. Agreement was low across both expertise groups and significantly lower at peripheral locations within a tumor than central locations. There was an interaction between expertise level and location within a tumor with experts displaying higher agreement at the peripheral slices than novices. An effect of brain image set on the order in which participants inspected the slices was also observed. The implications of these results for the training undertaken by early career radiologists and current practices in hospitals are discussed.

## Introduction

Brain tumors are the consequence of abnormal and uncontrollable cell growth in the brain and lead to deficits in the functioning of the body (Soltaninejad et al., 2015). Malignant brain tumors are cancerous, fast-growing and spread to other areas of the brain and spine whilst benign brain tumors grow more slowly and typically do not spread. In 2016, an estimated 77,670 primary brain and central nervous system (CNS) tumors were expected to be diagnosed in the United States (Ostrom et al., 2015). Clearly, accurate and reproducible detection and delineation of any brain tumor is essential for early diagnosis, patient monitoring, and treatment planning (Wu et al., 2013). Unhealthy brain tissue (i.e., tumor, necrosis) must be segmented from healthy brain tissue (i.e., gray and white matter, cerebrospinal fluid) and edema (Gordillo et al., 2013) to facilitate, where required, the surgical removal of a tumor, irradiation of the tumor bed, and monitoring of tumor growth or shrinkage (Mazzara et al., 2004; Bauer et al., 2013).

Despite considerable advances in computerized tools for brain tumor delineation (e.g., McBain et al., 2014; Weizman et al., 2014; Joshi et al., 2015) clinical acceptance of such tools has not yet been obtained. Therefore, in most clinical settings clinicians are responsible for the delineation of brain tumors. Particularly with infiltrative tumors, this task is time-consuming and challenging due to variations in tumor size, shape, and location. Clinicians must develop perceptual expertise to extract critical visual information from an image and then apply their clinical knowledge to successfully delineate a tumor (Porz et al., 2013). Brain tumor delineation is therefore constrained by clinician’s idiosyncratic perceptual and cognitive capabilities and thus the focus of this article is on better understanding where clinicians may differ from one another and non-clinicians in the delineation of healthy and unhealthy brain matter.

Magnetic resonance imaging (MRI) is widely used in delineation of brain tumors (DeAngelis, 2001; Wen et al., 2010) with 582,905 scans performed in the United Kingdom in 2014/15 (NHS England, 2015). This non-invasive technique generates images of comparable resolution to computed tomography (CT) using a strong magnetic field instead of the ionizing radiation used in CT and so is harmless to patients (Bhadauria et al., 2013). Different pulse sequences can be applied which enable the enhancement of specific tissues and so facilitates delineation (Bhadauria et al., 2013; Wu et al., 2013). In addition, since the MRI signals penetrate bone as well as soft tissue it is ideal for brain investigation (Islam and Hall, 2016). Manual outlining of gross tumor volume (GTV) is typically done on a slice-by-slice basis whereby clinicians scroll through a stack of 2D images reflecting the 3D volume of the brain, using sophisticated graphical user interfaces (Porz et al., 2013). Research is therefore needed to better understand performance in 3D dynamic stack-viewing of medical images which better characterizes the real-world (Ravesloot et al., 2015; Nakashima et al., 2016) and is fundamentally different to visual search in static medical images (Kunar and Watson, 2011). Moreover, given the extensive use of MRI scans in brain tumor delineation, more research is required to understand how clinicians delineate brain tumors using this specific modality.

Many studies have examined the degree of agreement between clinicians (i.e., inter-observer) in tumor delineation tasks. In a typical study, clinicians are asked to delineate (i.e., draw a boundary) around any tumorous tissue on a given slice or series of slices. The area on which two clinicians agree (i.e., they draw around the same tissue) is commonly referred to as the intersection area. A concordance rate (note: researchers refer to this value differently but it reflects the same calculation) ranging from 0% (complete disagreement) to 100% (complete agreement) can be calculated by dividing the intersection area by the sum of the area contoured by both clinicians (Murakami et al., 2012) to index inter-observer agreement. Leunens et al. (1993) reported that the concordance rate of 12 radiation oncologists delineating 5 patients’ brain tumors on lateral orthogonal radiographs ranged from 25 to 73%. Low inter-observer agreement was also observed amongst nine physicians delineating five patients’ supratentorial inoperable brain tumors on CT scans (range 38–59%) and on CT combined with MRI scans (range 38–71%; Weltens et al., 2001). Murakami et al. (2012) revealed an average concordance rate of 82% for one radiation oncologist and one neuroradiologist delineating glioblastomas using diagnostic MR images whilst Mazzara et al. (2004) reported an average concordance rate of 28% for three radiation oncologists outlining gliomas on MR images. Researchers have also revealed high intra-observer variability with Mazzara et al. (2004) reporting only a 20% concordance rate for three radiation oncologists delineating tumors at three 1-month intervals.

Inter-observer variability has also been documented in measures used to monitor tumor growth or shrinkage. Bidimensional product (BP) measurements (i.e., two perpendicular measurements in the largest area of the contrast-enhancing tumor) are used to assess the response of brain tumors to (and thus effectiveness of) radiation therapy. Complete agreement on the stability of a tumor for eight radiologists measuring brain tumor diameters in MRI scans was only observed in 45% of cases thus resulting in different judgements regarding the stability (growth or shrinkage) of a tumor (Provenzale et al., 2009). Provenzale and Mancini (2012) reported that in 76% of cases the same clinician changed their BP measurements by more than 25% (the criterion that indicates a change in tumor progression) between two examinations at 6–12 week intervals, which was deemed sufficient to prevent recall from the first measurement. This change in BP measurement resulted in a change in tumor stability judgements for 40% of cases (Provenzale and Mancini, 2012) and clearly demonstrates high intra-observer variability. Such research reveals high inter- and intra-observer variability and thus further research examining what underlies such disparities is clearly needed.

Only a few studies have investigated differences in tumor delineation across different expertise levels. Deeley et al. (2011) compared junior and senior physicians segmenting organs at risk in the brain and reported that junior physicians tended to segment volumetrically larger areas than their senior physician counterparts. Deeley et al. (2011) proposed that this reflected junior physicians’ tendency to avoid the risk of anatomically missing a portion of an organ and senior physicians’ confidence in delineating a tighter border. To our knowledge, no research has compared experts and novices in the delineation of brain tumors using MRI scans. Novice-expert paradigms have frequently been used to assess differences in the eye-gaze behavior of observers aiming to detect abnormalities in medical images to identify the perceptual and cognitive expertise required for successful performance in such tasks. Experts have been shown to have more efficient visual search strategies (Krupinski et al., 2006), fixate quicker on abnormalities (Kundel et al., 2007), and have superior global-processing advantages (rapidly extracting gross deviations from the norm; Evans et al., 2013). This paradigm can be applied here to identify differences in delineation and thereby identify techniques or strategies that facilitate precise and reproducible segmentation. By examining novices we have a comparison with which to establish the baseline level of performance in tumor segmentation from a relatively pure, perception-only perspective (i.e., with little or no background knowledge in tumor growth, localization, or geometry).

This study investigated the effect of expertise on the delineation of brain tumors using MRI images to identify the strategies or patterns of behavior that characterized expertise. Novices and experts freely inspected brain MRI images and were instructed to delineate tumorous from healthy brain matter. Using whole brains allowed us to capture a situation that is most similar to viewing in the real-world clinical situation where clinicians will scroll through a whole brain in order to detect a tumor. In addition to the whole brain series, we included a set of single slices from 16 different patients’ brains, to allow us to compare between whole brain volume delineation and single slice delineation. In line with Deeley et al. (2011), we anticipated that experts would delineate volumetrically smaller areas and delineate fewer slices than novices. Research indicating that experts delineate fewer slices may suggest greater disagreement at the peripheral locations of the tumor. We will therefore explore differences in inter-agreement at central and peripheral locations within the tumor. It is important to consider the order in which novices and experts examine and delineate tumors, especially when considering best practice to ensure optimum tumor removal and the problems associate with satisfaction of search. We therefore recorded the sequence of interactions participants made with each set of MRI scans and hypothesized that experts would engage in similar inspection techniques. We expected higher inter-observer agreement between experts given the specialized and similar training experts have undertaken and so their ability to apply both perceptual and clinical skill to the task. The effect of expertise on saliency-driven delineations and tumor-related differences in inter-observer agreement and inspection technique were also explored.

## Materials and Methods

### Participants

Twenty participants were divided into two groups based on their level of expertise in reading brain MRI scans. Novices consisted of undergraduate students at the University of Bristol, seven studying a non-medical subject and five studying a medical discipline (i.e., medicine, dentistry, veterinary science). Experts were eight radiologists at an NHS hospital (range of 2–15 years of experience). Ethical approval was gained from the Faculty of Science Human Research Ethics Committee at the University of Bristol. All participants gave written informed consent in accordance with the Declaration of Helsinki.

### Materials and Stimuli

**FIGURE 1:**
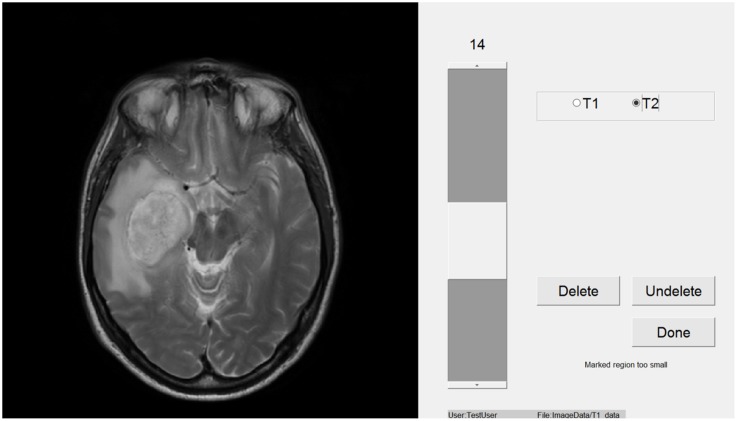
The viewing window on the tablet that participants used to complete the task.

**FIGURE 2:**
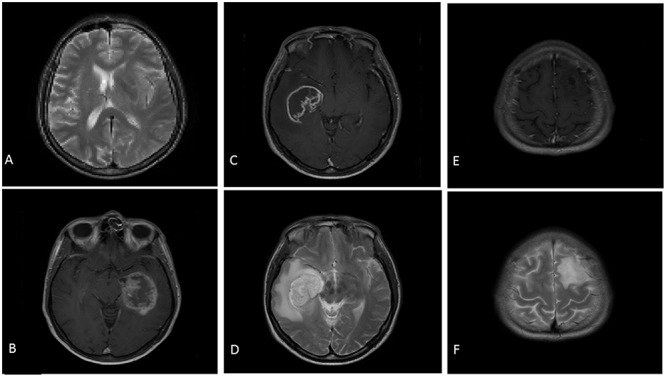
Examples of the stimuli presented to participants. **(A)** Is a slice from Set 1 (T2). **(B)** Is a slice from Set 2 (T1). **(C)** (T1) and **(D)** (T2) are slices from Set 3. **(E)** (T1) and **(F)** (T2) are slices from Set 4.

Participants viewed images within a window of a Toshiba Z10t Tablet PC with a diagonal screen size of 11.6 inch (1,920 pixels × 1,080 pixels resolution) and drew boundaries using a stylus (see **Figure 1**). Custom written software was used to display images and capture data using Matlab (The MathWorks, Inc., 2013). Set 1 consisted of images of 16 different brain tumors, each from a different patient. Eight of these were taken from a location in the center of the tumor and eight were taken from a peripheral location. **Table 1** shows the details of the MRI sets, because the sets were from actual clinical cases there are difference in their composition. Images were registered using SPM5 (Penny et al., 2001). **Figure 2** shows an example slice from each Set. Because the brains images were an opportunistic sample, we included an analysis by tumor type (or image set) as an exploratory analysis.

**Table 1:** Details of the stimuli in each image set.

Set	Slices	Anatomical plane	Imaging modality	Tumor description
1	16	Axial	T1 and T2	Various
2	31	Axial	T1	Left deep temporal posterior insular intrinsic tumor. This demonstrates ring enhancement following gadolinium. Histological diagnosis glioblastoma multiforme (grade 4)
3	31	Axial	T1 and T2	A right medial temporal lobe intrinsic tumor. This demonstrates ring enhancement following gadolinium. Histological diagnosis following resection was glioblastoma multiforme (grade 4)
4	31	Axial	T1 and T2	Intrinsic tumor within the left superior frontal gyms with signal heterogeneity. No enhancement demonstrated following gadolinium. Histological diagnosis following surgical resection was anaplastic oligoastrocytoma (grade 3)

### Design and Procedure

A mixed design with expertise level (novice; expert) as the between-subject factor and brain set (set 2; set 3; set 4) as the within-subject factor was used. To fully investigate inter-observer agreement, we also included location within a tumor (i.e., central vs. peripheral) as a within-subject factor. A mixed design was used to investigate the effect of expertise and location within a tumor on set 1 (i.e., independent slices). For the measure of Gross tumor area (GTA) location within a tumor was not included. Testing took place in various locations, but generally in a quite well-lit room. On arrival, participants were given instructions, and brief background details were recorded. Participants were then shown how the experimental software worked. Participants were given time to practice drawing around the border of some black and white high contrast familiar images (e.g., a car) using the tablet and stylus. Participants were then allowed to freely view each set of images and were instructed to draw around all tissue that they identified as tumorous on every slice. All participants moved sequentially through Set 1 to Set 4. Sets 2, 3, and 4 were whole brain scans so participants could navigate sequentially forward and backward from the superior to the inferior of the brain, respectively. Sets 3 and 4 consisted of both T1 and T2 images and so participants could switch between the same brain slice in these two modalities on which the same boundary was overlaid. Inspection and drawing time was not limited. Participants could view and delete any boundaries they had already drawn before moving onto the next set of images. The study took approximately 40 min to complete.

### Measures

#### Gross Tumor Volume

Gross tumor volume (GTV), defined as the sum of pixels delineated within an entire brain, was calculated for sets 2, 3, and 4. GTA was calculated for set 1 because all slices were from different patients’ MRI scans and so were independent of each other. This was calculated by dividing the sum of pixels delineated on all slices by the number of slices a participant drew a boundary on.

#### Number of Slices Delineated

The total number of slices on which a boundary was drawn when the participant submitted their response. Set 1 was not included in this analysis because every slice was from a different patient and all contained a tumor.

#### Inter-Observer Agreement

The intersection area, namely the total number of pixels that two participants delineated on a given slice was calculated. This area was then divided by the sum of the GTA contoured by the two participants to calculate a concordance rate ranging from 0% (complete disagreement) to 100% (complete agreement) used to index inter-observer agreement. This process was conducted between all participants within the same expertise group for each slice. Central locations within a tumor were the five slices on which most participants drew a boundary and so the highest agreement of detection of tumorous tissue was observed. Peripheral locations within a tumor were all other slices. For the whole brain sets concordance rates were then averaged across slices to provide each participants’ inter-observer agreement for a set.

#### ScanMatch Similarity Score

ScanMatch (Cristino et al., 2010) similarity scores were generated to statistically assess similarity in the sequence of experts and novices. If a participant engaged in two sequential interactions with a single slice then this slice was only included once in the sequence. The sequences of interactions were used to generate letter-string sequences which were then compared using ScanMatch which contains an implementation of the Needleman–Wunsch algorithm (frequently used, for example, to compare DNA sequences). A similarity score of 1 indicates that the sequences are identical whilst a score of 0 indicates that there is no similarity. Set 1 was not included in this analysis because all slices are independent of each other (i.e., from different patients) and so analysis of participants’ interactions was not relevant.

#### Saliency Driven Delineation

**FIGURE 3:**
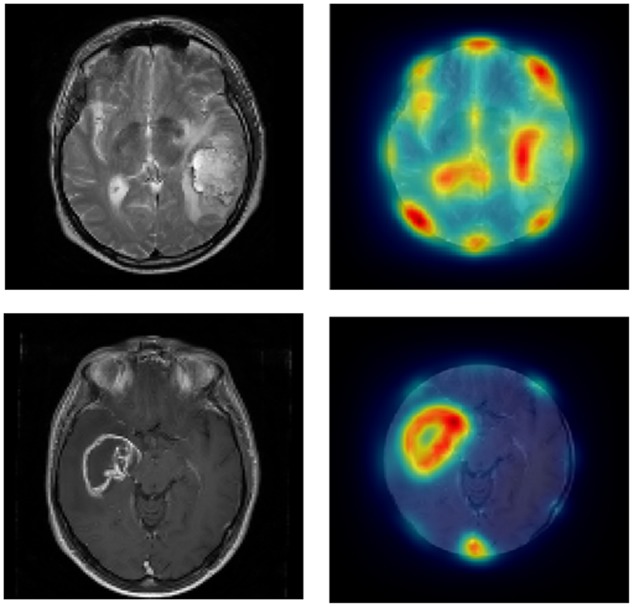
Left panels show the original images from Set 1 and the right panels show the same images cropped with saliency-based heat-map overlaid.

Saliency maps were generated using a graph based visual saliency Matlab implementation (Harel et al., 2006) for Set 1 (we manually removed the skull and eyes where they were visible, as these typically were highlighted as highly salient, and no observer ever drew around them). **Figure 3** shows an example of the saliency maps generated. The most, intermediate and least salient areas are shown in red, yellow, and blue, respectively. Each image is 512 pixels × 512 pixels with a value ranging from 0 (low salience) to 1 (high salience) representing the salience and so determining the color of a given pixel. We then multiplied the saliency map for each slice by the contour map (which has a 1 for any pixel the participant drew on the boundary and a 0 otherwise) for the corresponding slice and took the sum of the resulting matrix to give a summed contour-saliency score for each participant for each slice to index the extent to which delineation was saliency-based. Sets 2–4 were not included in this analysis because in these whole brain scans each slice is not independent and so delineation will be biased toward certain locations based on surrounding slices.

## Results

Linear mixed effects models (LMEs; Baayen et al., 2008; Barr et al., 2013) were used to analyze the data using the *lme4* package (Bates et al., 2013) for the R computing environment (R Development Core Team, 2014). All models include a random effect of participant. Additional random effects and interactions were only added when the more complex model fit the data significantly better according to a likelihood ratio test, in addition where a more complex model is used, we calculated the weighted Akaike’s information criteria (*w*AIC; see Wagenmakers and Farrell, 2004; we used the second order AIC, AICc, which is more suitable for smaller sample sizes). For all cases *w*AICc was approximately 1 for the more complex model and approximately 0 for the simpler model, demonstrating extreme evidence in favor of the more complex model. For each analysis, we report the *t* statistic from the full model (even if the model contained non-significant main effects and interactions) alongside *p*-values that were calculated using a model comparison procedure, for fixed effects and interactions of specific interest. For GTA, GTV and number of slices delineated we used a poisson distribution, since the data are count, using the *glmer* function in *lme4* and report *z*-value from the full model alongside *p*-values calculated using a model comparison procedure. Study participants did not consent to data sharing so supporting data cannot be made available.

### Main Analysis

#### Gross Tumor Volume

For the single brains, the more complex model including a random Group by Slice interaction was chosen over a model including a random effect of Subject and a random effect of Slice. For the full brain analysis, the model including a Brain by Subject random interaction was better than the model including only a random effect of Subject. There was no evidence for a difference in the GTA delineated by novices and experts for Set 1, *z* = 0.65, *p* = 0.517. There was also no effect of expertise on GTV delineation for Sets 2 – 4, *z* = 0.49, *p* = 0.629 (see **Table 2**).

**Table 2:** Descriptive statistics for gross tumor area (GTA) and gross tumor volume (GTV).

	Mean (pixels)		*SD* (pixels)	
	Expert	Novice	Expert	Novice
Set 1	6,364	6,261	1,862	2,700
Set 2	38,477	44,427	13,969	3,311
Set 3	35,320	50,761	11,920	47,698
Set 4	8,486	17,402	9,406	11,203

#### Number of Slices Delineated

**Table 3** shows that novices tended to delineate more slices than experts (there was never a case where an expert drew a boundary on a slice but a novice did not, whereas there were 31 cases where at least one novice drew on a slice that an expert did not) but this did not reach statistical significance, *z* = 0.28, *p* = 0.784.

**Table 3:** Descriptive statistics for number of slices delineated.

	Mean		*SD*	
	Expert	Novice	Expert	Novice
Set 2	7.63	9.08	1.60	1.24
Set 3	9.00	9.42	2.14	4.03
Set 4	4.43	7.50	2.15	5.23

#### Inter-Observer Agreement

For single brains, a more complex model including a random effect of Subject and a Group by Slice interaction was better than a model including a random effect of Subject and random effect of Slice. There was no evidence for a significant main effect of expertise level on inter-observer agreement for Set 1, *t* = 0.65, *p* = 0.517. There was evidence for a main effect of location on inter-observer agreement, *t* = 2.12, *p* = 0.046, with higher agreement observed at central locations that peripheral locations (see **Table 4**). There was no evidence for an interaction, *t* = 0.09, *p* = 0.926.

**Table 4:** Descriptive statistics for inter-observer agreement.

		Mean (%)		*SD* (%)	
		Expert	Novice	Expert	Novice
Set 1	Central	28.70	27.24	13.22	13.31
	Peripheral	15.89	10.86	12.20	10.62
Set 2	Central	33.63	38.68	5.69	3.85
	Peripheral	15.51	14.54	6.01	2.14
Set 3	Central	38.60	29.77	4.14	10.29
	Peripheral	11.29	6.89	3.33	2.39
Set 4	Central	10.64	26.31	5.47	12.60
	Peripheral	1.34	0.65	1.75	0.46

**FIGURE 4:**
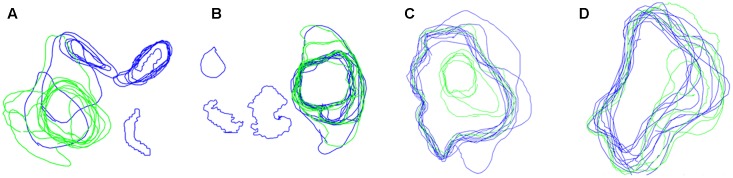
Boundaries drawn on peripheral locations within a tumor by Novices (blue contours) and experts (green contours), for four different brains **(A–D)**.

**FIGURE 5:**
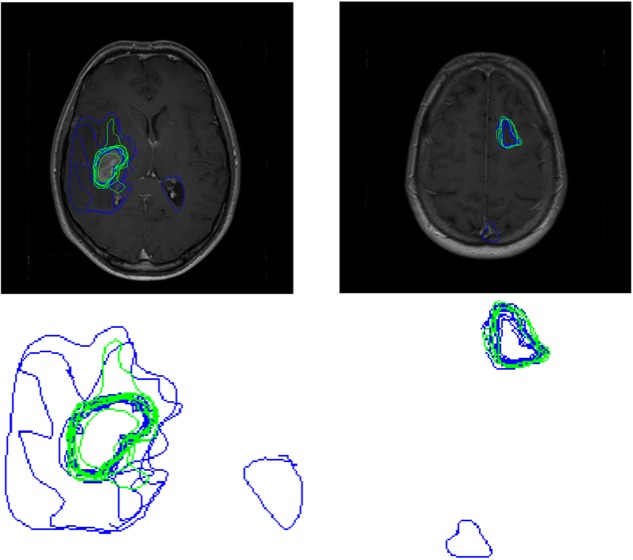
Boundaries drawn by Novices (blue contours) and experts (green contours) for a peripheral location within the tumor for Set 3 **(left)** and Set 4 **(right)**. The top row shows the images with participant boundaries. The bottom row shows an expanded view of the boundaries.

**FIGURE 6:**
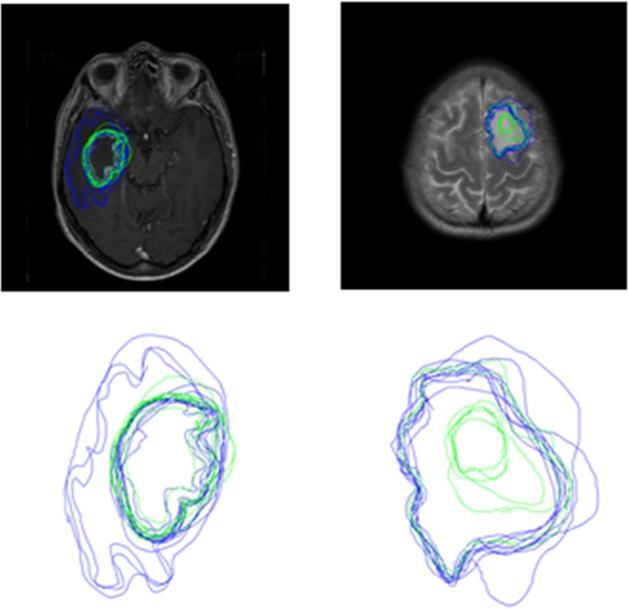
Boundaries drawn by Novices (blue contours) and experts (green contours) for a slice from Sets 3 **(left)** and Set 4 **(right)**.

**Figure 4** shows that agreement was low for both expertise levels. There was no evidence for a significant main effect of expertise level, *t* = 1.36, *p* = 0.176, or brain set, *t* = 0.67, *p* = 0.156, on inter-observer agreement. However, the location within a tumor did reliably influence inter-observer agreement, *t* = 0.86, *p* < 0.001 with agreement higher at central locations than peripheral locations. There was an interaction between expertise level and location with experts displaying higher agreement at peripheral locations than novices, *t* = 0.94, *p* < 0.001 (see **Figure 5**). There was also a significant interaction between brain set and group, *t* = 2.05, *p* < 0.001. Experts showed higher overall agreement for Set 3 but novices showed higher overall agreement for Set 4 (see **Figure 6**). A significant interaction between brain and location (*t* = 1.24, *p* < 0.001), was observed. There is a significant three-way interaction between group, brain and location, *t* = 1.58, *p* < 0.001. Novices agree more in the central locations within a tumor except for Set 3 where experts display higher agreement.

#### ScanMatch Similarity

**Table 5** shows that there was no evidence for a difference in ScanMatch similarity scores for novices compared to experts, *t* = 0.15, *p* = 0.882. There was, however, an effect of brain set on ScanMatch similarity, *t* = 2.14, *p* = 0.035, with set 4 showing the least similar patterns for both experts and novices. There was no significant interaction between brain set and expertise, *t* = 0.39, *p* = 0.700.

**Table 5:** Descriptive statistics for ScanMatch similarity score (higher is more similar).

	Mean		*SD*	
	Expert	Novice	Expert	Novice
Set 2	0.56	0.53	0.11	0.07
Set 3	0.54	0.42	0.11	0.11
Set 4	0.39	0.34	0.12	0.11

#### Saliency Driven Delineation

The model including a random effect of Subject and random effect of Slice was 1.30 times better than a more complex model with a random effect of Subject and random Group by Slice interaction. There was no evidence for a difference in saliency driven delineation between experts (*M* = 90.54, *SD* = 52.86) and novices (*M* = 95.60, *SD* = 55.61), *t* = 0.04, *p* = 0.968. There was, however, evidence for an effect of location on saliency-driven delineation, *t* = 3.90, *p* < 0.001. As expected, delineation at central locations (*M* = 101.64, *SD* = 53.06) overlapped more with salient areas than those at peripheral locations (*M* = 84.69, *SD* = 53.06). There was no significant interaction between expertise and location, *t* = 0.32, *p* = 0.746.

## Discussion

This was the first study to examine the effect of expertise on brain tumor delineation using MRI images. There was no evidence for a difference in the number of slices delineated between novices and experts. It is important to note, however, that there was not a single incident where an expert drew a boundary on a slice and a novice did not. This could reflect experts’ attempt to avoid the removal of healthy tissue and their superior ability to distinguish between different types of visual abnormality such as tumor or edema on peripheral locations within a tumor. In contrast to our hypothesis, and findings from Deeley et al. (2011), we did not find a reliable effect of expertise on either GTA or GTV. Although this is surprising, these measures are too simplistic to index successful performance on the task. We think in our task GTA and GTV capture information about the detection of a tumor but do not capture any information about the location of that tumor. It is possible, therefore, that a novice and an expert could draw entirely different regions of the brain on a given slice but still record the same score for GTA or GTV. Therefore, this finding does not necessarily indicate that the two expertise groups were performing in the same way which would be concerning given the extensive training that medical experts undertake.

We also investigated inter-observer agreement which captures information about the location of boundaries drawn by participants. In line with the existing literature (Leunens et al., 1993; Weltens et al., 2001; Mazzara et al., 2004), inter-observer agreement was low for both novices and experts. In contrast to our expectations, there was no main effect of expertise on inter-observer agreement. We propose that this is the result of high variability in the performance of both expertise levels and, more specifically, higher within-group than between-group variability. The low inter-observer agreement highlights the need for tools or practices that can facilitate a reduction in such variability. Eye-tracking research has revealed that viewing another person’s eye movements on a lung nodule detection task can improve detection performance (Litchfield et al., 2008, 2010). It is possible that a training practice where one views another person’s inspection technique through the slices and boundaries may improve overall performance. Double reading has been applied in mammography screening whereby two independent radiologists provide a reading with such practices leading to an increase in cancer detection rates from 5 to 17% (Helvie, 2007). The effect of double reading on success on brain tumor detection and delineation has not been studied and so research is needed to investigate the potential benefits of such practices.

Inter-observer agreement was higher for both expertise levels at the central compared to peripheral locations within a tumor. In line with the finding that delineation was driven more by saliency at central locations, we propose that saliency cues contributed to the higher agreement in delineation at central tumor locations. It is important to note that performance at central locations was far from perfect (or what may reasonably be considered as experts’ asymptotic level of performance) with the highest level of agreement for novices and experts being 38.68 and 38.60%, respectively. This demonstrates that there are high levels of disagreement as to where the tumor boundaries are, even when the tumor itself is relatively easy to detect. Experts displayed higher inter-observer agreement than novices in peripheral slices for the whole brain sets. We propose that, at peripheral locations, experts detect that there is tumorous tissue more often than novices and are better able to ‘see’ a boundary. It is also possible that experts learn to gain critical information from surrounding slices to inform their delineation of a tumor volume indicating that context may play an important role in this task. The role of context in other visual search tasks, such as finger print examination, has previously been documented (Busey et al., 2011). Taken together these findings reinforce the need to examine differences between static and dynamic medical image viewing.

Our results also revealed an interaction between brain image set and both group and location within a tumor. These results demonstrate that the characteristics of a brain tumor influence inter-observer agreement. Whilst we cannot make any strong conclusions from our data about which brain tumor characteristics specifically result in low inter-observer agreement, this finding highlights an important avenue for future research to identify types and locations of brain tumors that result in low agreement and so require additional training. For example, infiltrative tumors have hard to distinguish boundaries, which will make delineation more difficult. Wu et al. (2013) reported a difference in the accuracy of their semi-automatic segmentation methods for different brain tumors, suggesting that the characteristics of a brain tumor influence detection and delineation performance for humans as well as computer-based automated detection and delineation algorithms. The use of computer-based tools for diagnosis and delineation has grown rapidly over the last few years (Menze et al., 2015) but is still very much in development. Importantly, computer-based modeling can combine and use multimodal data, including substructure from diffusion tensor imaging, that are not so readily interpretable to a human observer, to finesse the tumor location (Alderson, 2016). Computer-based modeling can replicate the best practice and spread that knowledge to consistently identify the tumor and delineate the tumor boundaries to a higher standard across health services. One of the main impediments to their uptake is clinician and patient acceptance. Research should focus on demonstrating the potential improvement of human–computer cooperative delineation over just human-based delineation. What our data clearly show is that even experts sometimes disagree as to the precise location of a tumor boundary. This conclusion underpins the relatively poor prognosis for patients with brain tumors, and the need to research better delineation tools.

Using tumor delineation tasks, similar to the one used here, during training, combined with computer-based modeling, to identify cases that yield low inter-observer agreement could potentially reveal patterns in the type of and location of tumors that are particularly *difficult*. Training interventions have been reported to be effective in reducing variability in brain tumor delineation with Vinod et al. (2016) reporting a reduction in inter-observer variation following a teaching intervention in eight out of nine studies (although only four reached statistical significance). Breunig et al. (2012) also documented improvements in the segmentation of 10 out of 11 organs following education. Insight from studies similar to ours could further inform such interventions regarding the education provided to trainee radiologists. Moreover, identifying *difficult* cases could have implications for current practices, for example, making double reading or specialized training compulsory for certain tumor types. A potential limitation of our study is that we were unable to obtain both T1 and T2 imaging modalities for Set 2 and so, for this image, all participants had less information to drive their delineation. Interestingly, inter-observer agreement for Set 2 did not differ from the other Sets suggesting that this did not directly reduce inter-observer agreement. Investigating the effect of image modality on the delineation is an interesting avenue for future research.

We demonstrated a novel and effective application of ScanMatch to non-eye-tracking data which enabled investigation into the inspection technique adopted by participants. In contrast to our hypothesis that experts would engage in more similar inspection techniques as a result of following the standard practice for inspecting brain MR images learnt during their training (The Royal College of Radiologists, 2015), there was no evidence for a difference in inspection technique. A limitation of this measure, and potential explanation for why no significant difference was found, is that we did not record the time at which participants with a given slice to allow temporal binning of the data. This would have enabled the identification of slices with which a participant spent a long period of time interacting with and so provide more detail on disparities between the expertise levels. Inspection technique varied depending on the set of images inspected, which further demonstrates the need to examine the characteristic of tumors which lead to clinicians deviating from their typical inspection technique.

A limitation of the present study is the small sample size which leads to low power and increases the chance of obtaining false negatives (Button et al., 2013). We propose this may explain why statistical significance was not obtained in this experiment given the numerical tendency in the expected direction. This is, however, a common difficulty with such specialized research. Our expertise sample of eight radiologists is similar to the sample sizes used in existing research such as Copley et al. (2010) and Nakashima et al. (2016) who examined seven and ten radiologists, respectively. It is also important to recognize the high variation in boundaries which will potentially mask any subtle effects of expertise. Such large variation in the expertise group could be due to the range of expertise within our expert group (four radiology students; one certified radiologist; two consultant radiologists; one consultant neurologist). Statistical analysis could not be used to reliably investigate this difference in expertise due to a small sample size. Small samples are a common problem in medical image perception research, for example Copley et al. (2010) studied 4 certified radiologists and 6 radiology trainees. Nonetheless, given the importance of such research, detailed data sets such as ours are needed to start understanding the key factor involved before running later confirmatory studies. We also recognize that our novice sample, which includes students studying medically related and medically unrelated subjects at university, is quite varied which is a limitation of this research. Nevertheless, we believe it is difficult to find a truly ‘novice’ sample for this task (i.e., one without any prior exposure to MRI images) given the prevalence of MRI images in the media, but also the widespread use in various courses at university and we are confident that participants in the novice group had much lower levels of exposure than those in the expert group.

This study was the first to examine expertise-related differences in the delineation of brain tumors using MRI images. We also demonstrate the application of novel techniques, namely ScanMatch and saliency mapping, which will allow future studies to better characterize performance in these tasks. In line with existing research inter-observer agreement was relatively low thus further highlighting the need for improvements in consistency in brain tumor delineation across clinicians. Experts’ inter-observer agreement was higher than novices in the peripheral locations within a tumor. The effect of location within a tumor on inter-observer agreement suggests an important role for context in these tasks and demonstrates the need to better understand difference in delineation using static 2D compared to dynamic 3D images. Taken together our results have important implications for both training procedures and practices that may contribute to improved inter-observer agreement in clinical settings.

## Author Contributions

EC, WA, JR, and CK were responsible for the drafting of the report and analysis. EC, WA, and CK collected the data. WA, JR, and CK were responsible for the conception and design. JR was responsible for experimental software programming.

## Conflict of Interest Statement

The authors declare that the research was conducted in the absence of any commercial or financial relationships that could be construed as a potential conflict of interest.
